# A comparison of the effects of three luteal phase support protocols with estrogen on *in vitro* fertilization-embryo transfer outcomes in patients on a GnRH antagonist protocol

**DOI:** 10.5935/1518-0557.20190012

**Published:** 2019

**Authors:** Juliano Brum Scheffer, Bruno Brum Scheffer, Rafaela Friche de Carvalho, Ana Paula Aguiar, Daniel H. Mendez Lozano, Julie Labrosse, Michael Grynberg

**Affiliations:** 1 IBRRA – Brazilian Institute of Assisted Reproduction, Belo Horizonte, MG, Brazil; 2 School of Medicine, Tecnológico de Monterrey and Center for Reproductive Medicine CREASIS San Pedro Monterrey, México; 3 Department of Reproductive Medicine, Hôpital Jean Verdier (AP-HP), University Paris XIII; 4 INSERM, U782, Clamart - France

**Keywords:** luteal phase support, progesterone, estradiol, estrogen, pregnancy rate

## Abstract

**Objective:**

This study aimed to evaluate the effects of three different luteal phase support protocols with estrogen on the pregnancy rates and luteal phase hormone profiles of patients undergoing *in vitro* fertilization-embryo transfer (IVF-ET) cycles. A secondary objective was to evaluate which ovarian reserve markers correlated with pregnancy rates.

**Methods:**

This retrospective observational study was carried out at a private tertiary reproductive medicine teaching and research center. The study enrolled 104 patients undergoing intracytoplasmic sperm injection (ICSI) on an antagonist protocol for controlled ovarian hyperstimulation (COH). The women were divided into three groups based on the route of administration of estrogen (E2) for luteal phase support: oral (Primogyna); transdermal patches (Estradott); or transdermal gel (Oestrogel Pump). The administration of estrogen provided the equivalent to 4 mg of estradiol daily. All women received 600mg of vaginal progesterone (P) per day (Utrogestan) for luteal phase support. Blood samples were drawn on the day of hCG administration and on the day of beta hCG testing to measure E2 and P levels. Clinical pregnancy rate (PR) was the main endpoint.

**Results:**

The patients included in the three groups were comparable. No significant differences were found in implantation rates, clinical PR, miscarriage rates, multiple-pregnancy rates, E2 or P levels on the day of beta hCG measurement. Concerning ovarian reserve markers, significant correlations between testing positive for clinical pregnancy and AMH (r = 0.66*, p*<0.0001) and E2 levels on beta hCG measurement day (r = 0.77; *p*<.0001) were observed.

**Conclusions:**

No significant differences were seen in the pregnancy rates of patients submitted to IVF-ET cycles with GnRH antagonists given oral, transdermal patches, or transdermal gel E2 during the luteal phase. A correlation was found between clinical pregnancy rate and AMH and E2 levels on beta hCG testing day.

## INTRODUCTION

During the follicular phase of the menstrual cycle, estrogen (E2) plays an essential role in endometrial priming, as well as in the proliferation of uterine surface epithelium, glands, stroma, and blood vessels. However, the role of estrogen in endometrial preparation for embryo implantation during the luteal phase remains unclear, as some studies suggest that decreases in E2 during the luteal phase do not adversely affect the morphological developmental capacity of the endometrium (Younis *et al*., 1994; Pinheiro *et al*., 2017; Ismail Madkour *et al*., 2016). Steroids secreted in supraphysiological levels during the early luteal phase inhibit LH production, thus engendering low E2 and progesterone (P) levels (Hubayter & Muasher, 2008). Therefore, low E2 and P levels caused by a lack of luteal phase hormonal support in assisted reproduction technology cycles lead to decreased implantation and pregnancy rates (PR) (Hutchinson-Williams *et al*., 1989).

Despite the necessity of luteal phase supplementation to improve *in vitro* fertilization (IVF) outcomes (Nyboe Andersen *et al*., 2002), to our knowledge there is no consensus on the preferred type, dose, or timing of support. Although some studies described benefits from E2 supplementation (Lukaszuk *et al*., 2005; Gorkemli *et al*., 2004), others failed to observe positive impacts on support by E2 (Farhi *et al*., 2000; Ghanem *et al*., 2009; Smitz *et al*., 1993; Lewin *et al*., 1994; Tay & Lenton, 2003). Meta-analyses including these studies showed that supplementing P with E2 did not lead to better IVF outcomes (Serna *et al*., 2006; Jee *et al*., 2010). However, given the small size of these studies, larger series are required to determine the importance of E2 in luteal phase support, along with the most efficient dose and route of administration. To our knowledge, no publication has yet reported on the pregnancy effects of co-administering oral or transdermal E2 and progesterone in GnRH antagonist cycles.

This retrospective observational study compared the effects of three different luteal phase support protocols with estrogen on the outcomes of *in vitro* fertilization-embryo transfer (IVF-ET) cycles of patients on a GnRH antagonist protocol.

## MATERIALS AND METHODS

### Patients

This study included 110 women undergoing intracytoplasmic sperm injection (ICSI) at private reproductive medicine center Brazilian Institute of Assisted Reproduction (IBRRA) between March 2016 and February 2018. IVF-ET indications included tubal factor infertility, endometriosis, polycystic ovaries, normozoospermia, and unexplained infertility.

The inclusion criteria were as follows: i) both ovaries present; ii) no current or past diseases affecting the ovaries or the secretion, clearance, or excretion of gonadotropins or sex steroids; iii) patients could not be on hormone therapy at the time of treatment; iv) adequate visualization of the ovaries on transvaginal ultrasound examination; and v) small antral follicle (3-12 mm in diameter) count between 1 and 32 in the two ovaries added. Informed consent was obtained from all patients. The Institutional Review Board and the IBRRA Ethics Committee approved the study.

On oocyte pickup day, the patients were randomly assigned into one of three groups: transdermal estrogen gel daily (Group 1: Oestrogel pump, Estradiol- Besins Pharmaceuticals, Belgium); oral estrogen daily (Group 2: Primogyna - estradiol valerate, Bayer Pharmaceuticals, Germany); or transdermal estrogen patches daily (Group 3: Estradott- Estradiol, Novartis Pharmaceuticals, Switzerland) based on their application number. The researchers were blinded for treatment allocation.

### Treatment Protocol

Ovarian stimulation was performed with recombinant FSH (Gonal-F; Merck-Serono Pharmaceuticals, Italy), starting with a dose of 225-300IU on Day 2 of the menstrual cycle. When needed, FSH doses were adjusted starting from the fourth day of stimulation based on ultrasound findings and E2 blood levels. A GnRH antagonist (Cetrotide; Merck-Serono Pharmaceuticals, Italy) was administered at a dose of 250µg 0.5mL/day starting when the lead follicle reached 14-15mm in diameter, until the day of hCG injection.

Ovulation was induced by a subcutaneous (SC) injection of 250 mcg of recombinant hCG (Ovidrel, Merck-Serono Pharmaceuticals, Italy) when three follicles of at least 18 mm in diameter were observed on ultrasound examination. Oocyte pickup was performed 34 to 36 hours after hCG injection. Intracytoplasmic sperm injection (ICSI) was performed in all metaphase II oocytes. All patients underwent embryo transfer with ultrasound guidance on Day 3.

Supplementation with estrogen (transdermal gel, oral, or transdermal patches, according to randomization group) and intravaginal P 600mg once a day (Utrogestan, progesterone micronized, Besins Pharmaceuticals, Belgium) were administered to all patients on the day of oocyte retrieval. The three different administration routines of estrogen provided each the equivalent to 4 mg of estradiol daily. Blood samples were drawn on the day of hCG administration and on beta hCG measurement day (two weeks after ET), to measure E2 and P levels. Estrogen administration and intravaginal P were continued until pregnancy was ruled out by a negative serum beta-hCG test performed on day 14 after ET or until the twelfth week of pregnancy for pregnant patients. Clinical pregnancies were detected with the confirmation of a fetal heartbeat on transvaginal ultrasound examination. No drug-related side effects were reported in our study.

### Embryo Transfer Technique

ICSI was routinely performed in all fertilization procedures. Fertilization was evinced when two pronuclei were observed. Embryos were cultured until the day of transfer (Day 3) in IVF Global® media (Life Global, Canada) supplemented with 10% synthetic serum substitute (SSS) and graded based on the Veeck scoring system (Veeck, 1996) before transfer. The same embryologist performed all embryology procedures and embryo assessments in this study. All women received one or two embryos categorized as I and/or II. The definition over the number of embryos transferred was based on the guidelines of the Brazilian Federal Council of Medicine (FCM).

Embryo transfers were performed three days after oocyte retrieval. The patients were instructed to have a full bladder to provide for an acoustic window to visualize the uterus in preparation for the ultrasound-guided embryo transfer. Each patient was placed in the lithotomy position without anesthesia or sedation. The embryo transfers were performed with a Wallace Classic Soft Embryo Transfer Catheter, and abdominal ultrasound was performed using a 5 MHz probe (GE Logiq 400 Pro Series, General Electric Company, Pewaukee, WI).

### Laboratory methods and ultrasound scans

Serum AMH levels were measured using a second-generation enzyme-linked immunosorbent assay. Intra- and inter-assay coefficients of variation (CV) were <6% and <10% respectively, with a lower detection limit of 0.13ng/mL and linearity up to 21ng/mL for AMH. E2 and P levels were determined by electrochemiluminescence immunoassay (Elecsys and Cobas e analyzers; Roche Diagnostics GmbH, Mannheim, Germany). The results were determined via a curve specifically generated for the instrument by two-point calibration and based on the provided master curve. Sensitivity was 5pg/mL, and the linear interval of the test was 5 to 4,300 pg/mL for estrogen. E2 levels were determined with intra-assay and inter-assay coefficients of variation of <3.3% and <4.9%, respectively. Sensitivity was 0.21 ng/mL, and the linear interval of the test was 0.21 to 60ng/mL for P. P levels were assayed with intra-assay and inter-assay coefficients of variation of <8% and <9.1%, respectively.

Transvaginal ultrasound to assess the baseline antral follicle count was performed on Day 3 of the menstrual cycle. Follicles with a mean diameter of 3-12mm (mean of two orthogonal diameters) from both ovaries were considered. To optimize the reliability of ovarian follicular assessment, the ultrasound scanner was equipped with a tissue harmonic imaging system, which allowed for improved image resolution and adequate recognition of follicular borders. Intra-analysis CV for follicular and ovarian measurements were <5%, and the lower limit of detection was 0.1mm. In an effort to evaluate the bulk of granulosa cells in both ovaries, we calculated the mean follicle diameter (cumulative follicle diameter divided by the number of follicles measuring 3-12mm in diameter from both ovaries) and the largest follicle diameter.

This study aimed to evaluate whether the dose and mode of administration of estrogen affected the levels of estrogen on beta hCG measurement day and pregnancy rates, and which markers of ovarian reserve correlated with pregnancy rates. A secondary objective was to assess whether the levels of progesterone on beta hCG measurement day correlated with pregnancy rates.

### Statistical power calculation and statistical analysis

Statistical power calculation revealed that at least 30 patients were required in each arm of the study to attain significance in clinical pregnancy rates, the main endpoint analyzed in this randomized study. It was calculated for a difference of 25% in clinical pregnancy rates, as observed in the pilot study.

The level of significance (α) was 0.05 with a power of 0.95. The analysis of the number of clinical pregnancies showed that we had enough numbers to reach the required level of statistical power. Thus, enrollment was discontinued and the analysis of results commenced.

Data sets were analyzed on SPSS for Windows release 15.0 (SPSS, Inc., Chicago, IL). Continuous data were expressed as mean values ± SD. Data following a normal distribution were analyzed with one-way ANOVA, whereas the Kruskal-Wallis test was used for the remaining data. Categorical data were analyzed with Pearson’s c^2^ test. If statistical difference was found, the groups were compared by the c^2^ test with the Spearman correction. E2, P, and E2/P rates for ongoing pregnancies in all groups were analyzed with the Mann-Whitney U test. Significance was set at 5%.

## RESULTS

### Patient characteristics

Our retrospective study included 110 patients. Six patients were not present on the day of beta HCG measurement and were thus excluded. The final study population was 104. Group 1 had 32 patients, Group 2 had 33 patients, and Group 3 had 39 patients. Patient characteristics are described in Table 1. There was no statistically significant difference between groups for age, body mass index (BMI), Day 3 FSH and Anti-Müllerian hormone (AMH) levels, antral follicle count (AFC), length of stimulation, total dose of gonadotropin, or peak E2 and P levels on hCG injection day (Table 1).

**Table 1:** Patient and cycle characteristics for the three treatment groups

Characteristic	Group 1 (E2 gel)	Group 2 (E2 oral)	Group 3 (E2 patch)	*p*-value
n	32	33	39	
Age (y)	33.81±3.34	35.0±4.81	34.61±4.49	0.50
BMI (Kg/m^2^)	24.28±2.62	25.83±5.80	24.02±3.10	0.14
Day 3 FSH(mUI/mL)	10.37±6.80	12.51±7.63	12.71±8.41	0.38
AFC	13.53±4.64	12.06±4.58	13.12±4.57	0.41
Day 3 AMH (ng/mL)	3.50±3.66	2.82±2.59	2.75± 2.68	0.52
Length of stimulation (d)	10.00±1.66	9.75±1.52	10.61±1.51	0.06
Gonadotropin dose (IU)	1929.68±777.51	2041.51±691.47	2193.59±757.80	0.32
Peak E2 level hCG day administration (pg/ml)	2785.62±873.97	2315.00±984.49	2555.64±907.50	0.12
Peak P level hCG day administration (ng/ml)	0.39±0.26	0.42±0.27	0.52±0.27	0.09

Note: *p*<0.05 was considered to be statistically significant. Data are expressed as mean values ± SD

### Outcome of ART treatment

**Figure 1:**
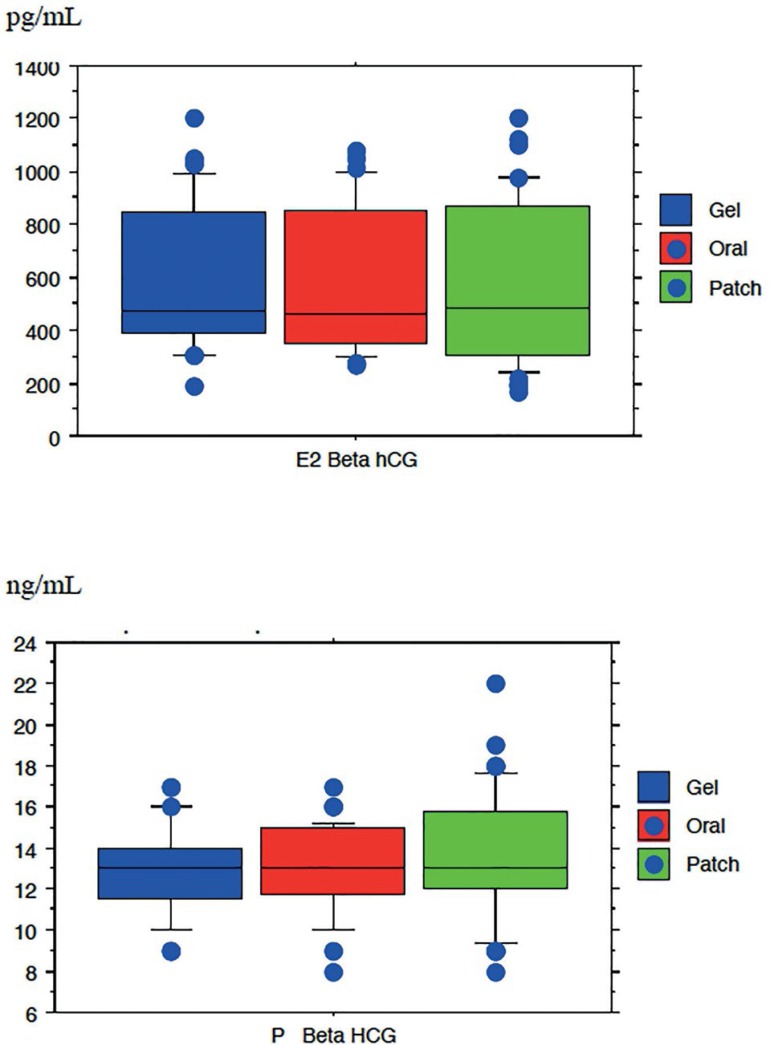
Level of estradiol (E2) and progesterone (P) on beta hCG measurement day of the three treatment groups (*p*>0.05)

No significant difference was found in the number of oocytes retrieved, number of embryos I + II, number of embryos transferred, implantation rates, clinical PR, miscarriage rates, multiple-pregnancy rates, E2 and P levels on beta hCG measurement day (Table 2) (Figure 1).

**Table 2:** *In vitro* fertilization-embryo transfer cycle characteristics of the three treatment groups

Characteristic	Group 1 (E2 gel)	Group 2 (E2 oral)	Group 3 (E2 patch)	*p*-value
N	32	33	39	
No. of oocytes retrieved	11.12±4.42	8.78±4.56	8.87±4.47	0.06
No. of embryos I+II	3.66±2.4	3.03±1.48	3.12±2.85	0.39
No. of embryos transferred	1.96±0.17	1.90±0.29	1.89±0.30	0.50
Implantation rate (%)	20.31±27.99	22.72±30.85	17.94±26.87	0.77
Clinical PR, % (no.)	43.7 (14/32)	42.4(14/33)	38.4 (15/39)	0.86
Miscarriage rate % (no.)	14.2 (2/14)	14.2 (2/14)	13.3 (2/15)	0.43
Multiple-pregnancy rate, % (no.)	3.12 (1/32)	3.03(1/33)	2.56 (1/39)	0.63
E2 level beta hCG day (pg/ml)	605.34±278.94	595.06±281.73	571.23±303.86	0.87
P level beta hCG day (ng/ml)	12.81±2.17	12.93±2.17	13.64±3.03	0.32
Δ E2 level beta hCG day E2 level hCG administration	2180.28±16.53	1719.93±12.14	1984.41±14.38	0.07
Δ P level beta hCG day P level hCG administration	12.41±3.33	12.51±3.51	13.11±2.69	0.08

Note: *p*<0.05 was considered to be statistically significant. Data are expressed as mean values ± SD or as proportions and absolute numbers. Δ – Mean of Variation of hormone profile

### Hormonal profile

There was no significant difference in the levels of E2 and progesterone on hCG injection day and ß hCG measurement day in the three groups (*p*>0.05). The E2/P ratio on beta HCG measurement day was comparable between the three groups, showing that the mode of administration of estrogen in the luteal phase did not lead to different effects on hormonal profiles (Table 3).

**Table 3:** Comparison of hormone profile variance in the three treatment groups

Variance rate of hormone profile	*p*-value		
	Group 1 x 2	Group 1 x 3	Group 2 x3
E2 hCG day / E2 beta hCG day	0.09	0.06	0.06
P hCG day / P beta hCG day	0.09	0.06	0.06
E2 beta hCG day / P hCG day	0.08	0.08	0.09

Note: *p*<0.05 was considered to be statistically significant

### Clinical Pregnancy

**Figure 2:**
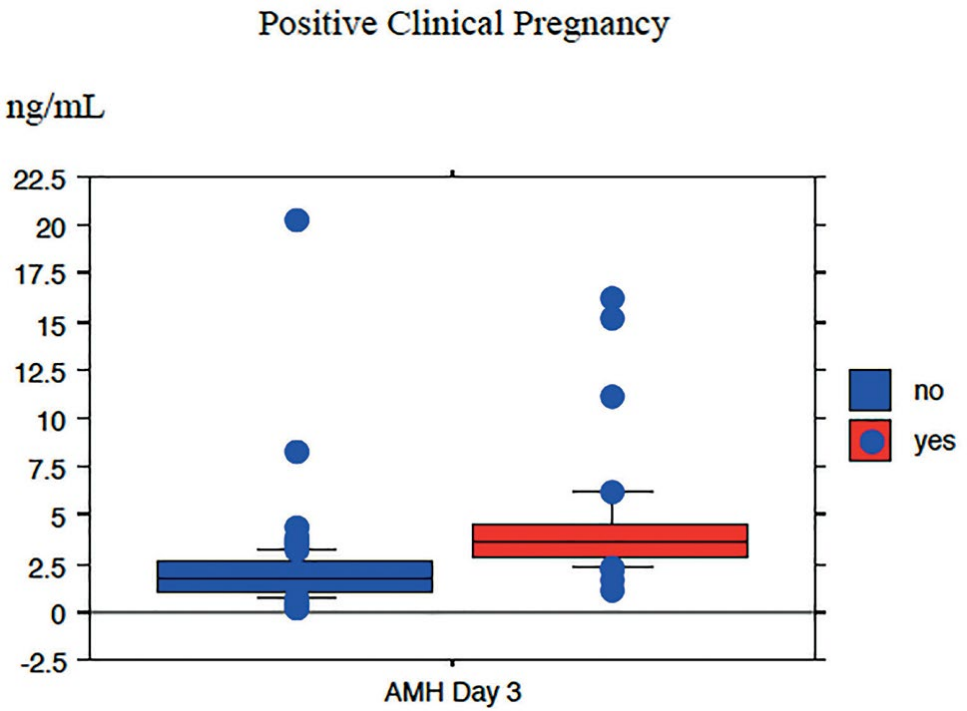
Spearman’ s correlation of positive clinical pregnancy test and AMH level (*p*< 0.0001)

**Figure 3:**
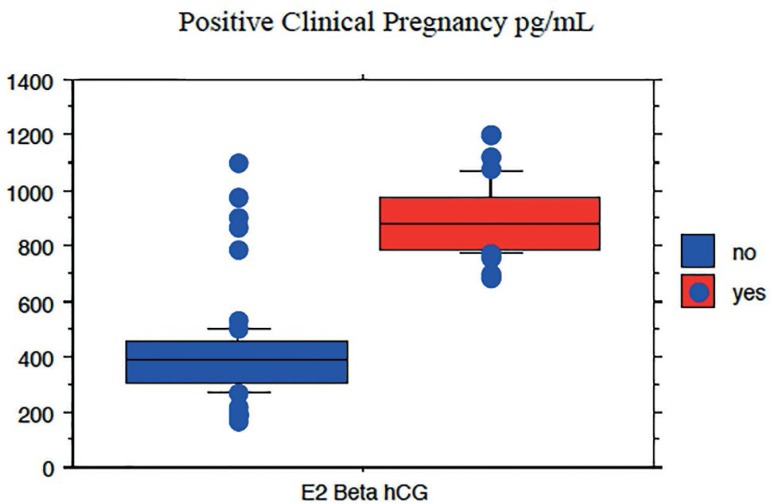
Spearman’ s correlation for positive clinical pregnancy tests and E2 levels on beta hCG measurement day (*p*<0.0001)

In terms of ovarian reserve markers, a significant correlation was observed between testing positive for clinical pregnancy and AMH levels (r=0.66*, p*<0.0001) (Figure 2). In relation to the hormonal profile, positive pregnancy tests were significantly associated with E2 levels on beta hCG measurement day (r=0.77 *p*<0.0001) (Figure 3), regardless of the estrogen protocol chosen for luteal phase support. Concerning the variables significantly correlated with positive pregnancy tests, the median E2 level on beta hCG measurement day was 903.65±127.85pg/mL and the median level of AMH on Day 3 was 4.43±3.14ng/mL, regardless of the estrogen protocol chosen for luteal phase support. Thus, the mode of administration of estrogen in the luteal phase did not affect the outcome of ART treatment.

## DISCUSSION

Based on the findings from a cohort of 104 patients, our retrospective study showed that in IVF-ET protocols including cycles with a GnRH antagonist, the use of oral medication, transdermal patches, or transdermal gel during the luteal phase did not significantly affect pregnancy rates. To our knowledge, our study was the first to compare the effects on IVF-ET cycle outcomes of three different luteal phase support protocols with estrogen in patients given a GnRH antagonist.

Embryo implantation is a complex dynamic process that involves structural and morphological changes to the embryo and the endometrium. Adequate levels of estrogen and P may be essential for optimal endometrial maturation before embryo implantation, as a lack of synchrony between the stages of embryo development and endometrial maturation may result in implantation failure. The idea that E2 might be used throughout the luteal phase in IVF cycles emerged when Smitz *et al*. (1988) showed that serum E2 concentrations dropped at the end of the luteal phase. Cycles using GnRH agonists and antagonists have been associated with poor luteal phase hormonal production. Although the role of P supplementation in the luteal phase of down-regulated cycles is well established, there have been only a few attempts to clarify the benefits of adding E2 therapy in these cycles. The use of E2 during the luteal phase, including its role in the preparation of the endometrium for implantation, remains rather controversial.

Stewart *et al*. (1993) were the first to identify a significant difference in serum E2 levels between conception and non-conception cycles in fertile women undergoing donor insemination. This difference was noted as early as Day 6 after the LH surge. Similarly, a rise in luteal E2 on Day 6 in conception cycles compared with non-conception cycles was found in a group of 32 women trying to conceive spontaneously (Baird *et al*., 1997). Other studies have reported an association between elevated and steadily increasing serum E2 levels in the luteal phase of IVF-ET cycles and higher PRs (Emperaire *et al*., 1984). Subsequently, Sharara & McClamrock (1999) revealed that the magnitude of the decline in serum E2 concentrations, measured by the ratio of peak E2 (on the day of hCG administration) to midluteal E2 (10 days after hCG administration), was predictive of IVF success. A sharp decline in midluteal E2, defined as a peak E2 to midluteal E2 ratio greater than 5, resulted in significantly lower implantation and OP rates. All of the above data raised the issue around a potential positive correlation between elevated E2 levels in the luteal phase and conception, in addition to the need to elucidate the relationship between estradiol on the day of beta hCG measurement and pregnancy, as seen in our study. Elgindy *et al*. (2010) found the lowest E2 levels on Days 7, 10, and 13 in the P-only group during the luteal phase, and further showed that the decreases on days 7 and 10 were the highest. A dose-finding RCT (Lukaszuk *et al*., 2005) reported that the best implantation and pregnancy rates were recorded in the group given 6 mg E2 supplementation compared with 2 mg or no E2 supplementation. Regarding the route of administration, most of the previous studies used the oral route (Lukaszuk *et al*., 2005; Farhi *et al*., 2000; Smitz *et al*., 1993; Lewin *et al*., 1994; Fatemi *et al*., 2006; 2007)

In our study, the three groups did not differ in regards to maximum E2 levels on the day of hCG administration. Addition of E2 (oral or transdermal) did not significantly change the endocrine profile of the luteal phase. Although Lewin *et al*. (1994) reported no significant difference in luteal E2 levels upon oral supplementation with 2 mg E2 valerate, Farhi *et al*. (2000) reported significantly higher E2 levels in non-conception cycles on Days 11, 14, and 16 after hCG administration upon supplementation with 4 mg E2 valerate. Fatemi *et al*. (2007) observed that the addition of 4 mg E2 valerate to P for luteal phase support in antagonist cycles did not affect the E2 level significantly until Day 10 after hCG administration, when it was associated with significantly higher E2 levels. However, the effect of higher levels of E2 on the endocrine profile could not be ruled out. Morphologic studies have demonstrated that the endometrium is sensitive to decreases in steroid levels and subnormal midluteal E2 concentrations (24). During the luteal phase, estrogen has a modulatory effect on the secretory endometrial P receptor concentration and may serve to replenish and maintain a requisite level of P receptors to mediate and complete the P response (Fritz *et al*., 1987; Goldstein *et al*., 1982)

There is no consensus regarding the optimal dose and duration of E2 administration during the luteal phase. A well-conducted randomized trial (Hutchinson-Williams *et al*., 1989) looked into the effects of different E2 supplementation doses on IRs and PRs using the long GnRH-a protocol. All patients received P4 vaginally (600 mg/day) and were randomly allocated to daily doses of 0, 2, or 6mg of E2. Significantly higher IRs and PRs were recorded in patients given low-dose E2 supplementation compared to those who did not. The subgroup meta-analyses on different doses of E2 suggested similar trends toward favorable outcomes in the group given a combination of E2 and P4, but the number of studies was very limited, precluding the extraction of clear conclusions regarding optimal E2 doses. These discrepancies may be attributed to the different methodological designs across studies. Further studies are required to determine the role of luteal E2 supplementation in IVF and investigate its optimal regimen (dose and route).

Although our study showed a significant association between testing positive for clinical pregnancy and AMH levels, ovarian reserve markers reportedly have some predictive power in the realm of assisted reproductive technology (ART) treatments. However, there is consensus that they provide only general approximations of stimulation quantity (e.g., the number of oocytes retrieved in ART treatment cycles). The main limitations of these tests include their poor sensitivity and, in most cases, their dependency on cycle stage. Furthermore, once a woman tests abnormal, poor prognosis is assigned to her ART treatment possibilities (Scheffer *et al*., 2017)

In our study, no significant difference on pregnancy rates was observed between the administration of oral estrogen, transdermal estrogen patches, or transdermal estrogen gel as luteal phase support in IVF-ET GnRH antagonist protocols. Further research in this area is warranted to confirm and advance these findings.

## CONCLUSION

In IVF-ET cycles with a GnRH antagonist, no significant difference was observed on pregnancy rates when patients were given oral E2, transdermal E2 patches, or transdermal E2 gel during the luteal phase. Clinical pregnancy rates correlated with AMH and E2 levels on beta hCG measurement day.
